# Consumer transparency in the production chain for plant varieties produced using new genomic techniques

**DOI:** 10.1007/s42994-024-00142-y

**Published:** 2024-04-01

**Authors:** J. M. Lukasiewicz, C. C. M. van de Wiel, L. A. P. Lotz, M. J. M. Smulders

**Affiliations:** Wageningen University & Research, P.O. Box 386, NL-6700 AJ Wageningen, The Netherlands

**Keywords:** Labelling, New genomic techniques, Consumer transparency, Plant products

## Abstract

Plants edited with new genomic techniques (NGTs) currently fall under the Genetically Modified Organisms Directive (2001/18/EC) in the European Union. In the proposal of the European Commission, NGT plants are partially exempted from the regulations of this directive. The proposal makes a distinction between two categories of NGT plants: NGT-1 and NGT-2. NGT-1 category plants are considered equal to plants obtained through conventional breeding methods. These plants will not be labelled for the consumer, although they will be labelled as seeds. NGT-2 category plants may be labelled with additional information as a positive incentive. Labelling of seeds of varieties made with gene editing, but not the products, would mean that most steps in the production chain are transparent, but not the last step towards consumers. The “right to know” and increasing knowledge of gene-edited food is a common theme in food labelling towards consumers. Here, we describe current labelling regimes and registers and how these may be applied to provide transparency on gene-edited products to consumers. Furthermore, we also look into consumer studies, which indicate a greater acceptance of gene-edited food among consumers, especially when additional benefits such as sustainability are mentioned.

## Introduction

In a 2018 ruling of the European Court of Justice, plants derived from new genomic techniques (NGTs) were fully placed under the Genetically Modified Organism (GMO) Directive of 2001/18/EC and have to comply with, notably, Regulation (EC no 1830/2003) on traceability and labelling and Regulation (EC 1829/2003) on genetically modified food and feed. After a study followed by public consultations, the European Commission concluded that the 2001/18/EC GMO Directive was not fit for purpose anymore, and a proposal for a new regulation was developed.

In the European Commission’s proposal on NGTs (European Commission 2023b), gene-edited (GE) varieties in the NGT-1 category, which contain a limited number and type of modifications, are considered equivalent to conventional plants and do not require separate labelling for consumers. These varieties will only be listed in a public register, whereas seed and plant reproductive material (PRM) will be labelled to provide information to actors in the production chain. NGT-2 category varieties, which contain more and/or larger modifications compared to NGT-1, will remain labelled according to GMO Directive (2001/18/EC) and Regulation (EC no 1830/2003) (European Commission 2001b), with the additional possibility of adding the modified trait and its benefits as a positive incentive to the label.

With regards to transparency, NGT-1 is, according to the proposal, a new category as plants of this category will not fall under current obligatory labelling requirements. Critics (e.g. Foodwatch (Foodwatch 2023)) of the NGT proposal use the argument that customer choice is not guaranteed, despite the existence of a (future) public register for NGT-1 varieties and the mandatory labelling for (sowing) seed producers in the production chain. In their view, NGT-1 category plants should also contain a label specifying them as genetically modified for consumers.

This review presents the current mandatory and voluntary labelling landscape in the European Union (EU) and provides insights from consumer studies, which may be relevant to positive and negative labelling options of NGT products in view of changing consumer acceptance. It also provides and discusses insights on what a possible NGT registration may look like, based on current lists and registers.

### The current labelling landscape for consumers

Food labelling primarily serves to provide transparency for informed freedom of choice for consumers, but also for actors in a production chain. The FAO report on Environmental and Social Standards, Certification and Labelling for Cash Crops contains a chapter with an overview of several standards and certification programmes for food, set by ISO (International Organization for Standardization) (Dankers 2003). Standards may be obligatory, such as national food safety standards that are based on guidelines set by intergovernmental bodies (e.g. the FAO/WHO Codex Alimentarius Commission) or voluntary in the sense that one can choose not to carry the label. When using the label, one must always comply with the corresponding regulation.

A label can refer to the contents of the products (e.g. sugar, protein, salt, presence of E-number additives, presence of allergens), to characteristics of the type of production (e.g. organic), to characteristics or minimum requirements in the production chain (e.g. Fairtrade, Marine Stewardship Council (MSC), or Roundtable on Sustainable Palm Oil (RSPO)), or to marketing concepts. Often a label does not just provide neutral information but instead refers to something that may be attractive to people (positive labelling such as sustainability or animal welfare) or something one may want to avoid (negative labelling such as dietary components or allergens). Under EU law (European Commission 2011), the following information is, among others, required for food packaging: ingredient list, allergen information, and country of origin. Ingredient lists are not required for foods sold as a whole, such as fruit and vegetables.

An example of national voluntary labelling is the non-GMO label (Enga 2023). The non-GMO label also excludes food products such as eggs and milk directly derived from animals that were fed GMO feed; these products are currently not labelled under EU law. According to the European Non-GMO Industry Association, some countries (such as the Netherlands, Belgium, and Sweden) have regulations restricting the use of non-GMO labels (Enga 2023). However, in the Netherlands, a label saying that no GM techniques have been used is allowed, although, since three food categories (conventional, organic, or GMO) have already been defined, a non-GMO label does not provide additional information (Pinckaers 2019), except in cases of animal products where genetically modified (GM) feed has been used. In contrast to GE products, in which small genetic modifications have been made or cisgenes inserted, GM products typically contain foreign DNA in the form of one or more transgene(s).

### GMO labelling

Next to required information (including allergens) for all food packages and voluntary labelling, a GM label is mandatory at EU level if a product is or contains a GMO or products derived from it (above the threshold of 0.9%). Contrary to popular conceptions, GMOs are allowed to be cultivated and sold within the EU. However, they are subject to a thorough and costly risk assessment (European Commission 2001a), a process which only large companies undertake due to the high costs and long duration. Currently, only one GMO event (MON810 Bt maize) is grown within the EU (in Spain and Portugal). Some EU countries have stopped the cultivation of GMOs altogether (Romania, the Czech Republic, and Slovakia) and/or have since banned GMO cultivation on their soil (Genetic Literary Project 2023). Fifteen EU countries have banned GMO cultivation by 2015, following a legislative adaptation of the Directive on the EU level (Directive 2015/142). At the same time, a large number of GMO events have been approved for import for food (108 events) and feed (109 events) as direct use or as an additive, and GMO varieties with these events are imported into the EU in large quantities (Agricultural Biotechnology Journal, 2018). A GMO label on pre-packaged products contains the words “genetically modified” or “produced from genetically modified (and the name of the organism)” (European Commission 2001c).

In the USA, labelling of GMOs was not mandatory, but as of 1 January 2022, US food packaging requires the label “bio-engineered” or “derived from bio-engineering” when GM techniques have been used in the breeding process. A study by the National Research Center (2014) showed that most US consumers (92%) prefer to have their food labelled. However, it is not expected that the new bio-engineered label will affect consumer choice since consumers who want to avoid GMO food already did so before the implementation of the label (Adalja et al. 2023). This labelling does not include GE plants, as products derived from GE that are similar to conventional plants have been placed outside of regulatory supervision by USDA-APHIS (Animal Plant Health Inspection Service) (Kuzma and Grieger 2020; Lindberg et al. 2023). Similarly, in Canada, bio-engineered varieties that are not “novel” (i.e. food that is not new or changed compared to existing foods) do not require labelling (Government of Canada 2023). Instead, a voluntary database exists where manufacturers can list all their varieties, both conventional and bio-engineered.

In the EU, consumer concerns about GMOs have dropped over the years. According to the Eurobarometer, the number of EU citizens concerned about the presence of GMOs in the environment decreased from 30% in 2002 to 19% in 2011, while concern about the use of GM ingredients in food and drinks dropped from 63% in 2005 to 27% in 2019 (Ichim 2021). In terms of labelling, nobody is negative about transparency. The greater part of consumers support, when asked, labelling and thus prefer to be directly informed about their food choices. It remains to be seen, however, whether an abundance of information might lead to consumer stress in making informed decisions.

### NGT transparency through registers and lists

In the current NGT proposal of the European Commission, NGT-1 varieties would not be labelled, while NGT-2 varieties remain labelled under the GMO Directive (2001/18/EC) with the possibility of adding the modified trait to the label. NGT-1 varieties will be listed in a public register, while seed and PRM remain labelled in the production chain, thus enabling growers, producers, and retailers to choose between NGT or non-NGT varieties.

In the UK, the British Society of Plant Breeders (BSBP) has previously announced that it will establish a similar public register of crop varieties based on NGTs to increase transparency in the supply chain (BSPB 2022). This is part of the Precision Breeding Bill in the UK, a proposition for an adapted regulatory regime for NGTs that lead to products that are similar to those from conventional breeding. The proposition is comparable to how jurisdictions elsewhere in the world, including the USA and Canada, deal with plants produced with NGTs. In the Netherlands, Plantum, the trade association of plant breeding and propagation companies, has also advocated transparency by registering the use of NGTs in plant breeding products (varieties) to provide freedom of choice, in a recent round table discussion at the Dutch Parliament (Plantum 2023).

In organic cultivation and organic breeding programmes, so-called positive lists with vegetable varieties already exist. These lists are used to exclude varieties in organic cultivation, e.g. varieties in which cytoplasmic male sterility (CMS) has been used (Lammerts Van Bueren et al. 1999). Another example of a positive list is a list with cell-fusion-free varieties (FiBL 2022). These lists enable organic growers to select varieties that are free of a certain type of breeding technique that is not in line with organic principles for cultivation employed in certain types of organic or biodynamic agriculture.

Next to the principles of Distinctness, Uniformity, and Stability (DUS) of the International Union for the Protection of New Varieties of Plants (UPOV) guidelines, new varieties can also be tested for *Value for Cultivation and Use* (VCU) which is a long-standing institutional way of promoting sustainability of cultivations for arable crops. Only varieties on this list are allowed to be cultivated in the EU and are included in the EU common catalogue of agricultural crops (European Commission 2023c). The new variety needs to show clear improvement in traits for cultivation (e.g. yield), use, or the harvested product quality for the targeted region of cultivation as compared to varieties already on the list (Van de Wiel 2002). VCU does not exist for vegetables, fruits, and ornamental crops.

The European Commission also proposed a revision of the PRM and FRM (forest reproductive material) legislation this year. This is meant to update and modernize the current PRM regulation, and the basic principle of variety registration and PRM certification will remain the same. Similarly to the previous VCU list, new varieties of arable crops, vegetables, and fruit that conform to DUS will have the option to be included on a list of *Value for Sustainable Cultivation and Use* (VSCU) which promotes a more sustainable approach (European Commission 2023a). Registered varieties in any national catalogue will be accessible on the EU Plant Variety Portal. A similar system could be used for NGT-1 varieties. Indeed, Dima et al. (2023) mention the use of the European Commission’s common catalogue of agricultural crops as the database for NGT varieties as well.

### Consumer preferences regarding GE labelling

The basic idea is that labelling supports the consumers’ freedom of choice. As pointed out above, mandatory labelling is aimed at allowing consumers to make an informed decision about their diet, e.g. for certain health effects or allergenic effects, while voluntary labelling generally conveys positive connotations such as animal welfare or sustainability. GM labelling takes a unique position, since it does not convey information on diet, safety, or sustainability, but instead mentions the technology used. Indeed, bio-engineering proponents argue that labelling of GM products is not informative, since it would not be related to food safety (or quality) as these products have already gone through regulatory scrutiny. In addition, separate GM labelling would unnecessarily raise costs to enact and monitor labelling for all consumers, including those that do not oppose GM food (Kalaitzandonakes et al. 2018).

GE makes use of NGTs, which were developed since the first (transgenic) GM crops, to precisely edit specific genes. Therefore, GE food may lead to other consumer perceptions than GM food. Agronomic traits such as pest or pathogen resistances generated using NGTs can be positively labelled, which could form an attractive marketing claim. However, these benefits should be clear towards consumers, since traits solely benefitting the producer or difficult-to-judge benefits do not lead to higher acceptance (Mather et al. 2012). In a survey using GE table grapes, the results suggested a higher willingness of consumers to pay for improved taste and texture than for a lower level of pesticide applications (Uddin et al. 2022). The same was observed in GE potatoes, where consumers required a smaller price discount for potatoes with health-related improvements (such as lower acrylamide) compared to potatoes with environmentally related improvements (such as lower pesticide and water usage) (Muringai et al. 2020).

A study by Ferrari et al. (2021) indicated an overall positive attitude towards GE food among 234 respondents in the age range of 18–38 years in the Netherlands and Belgium. However, consumers with concerns about the environment tended to be more critical of GE food. Thus, environmental concern was not found to be a relevant factor in preference for a positive label for GE, even though there are GE applications that may provide lower environmental impacts of crop cultivation. This could point to a lack of awareness of these potential environmental effects (Ferrari et al. 2021). The same study also found an effect of higher demonstrable knowledge on attitudes towards GE food, which negatively affected the preference for a separate GE label. The authors noted that the effects of levels of knowledge on consumer preferences may not be generalizable to all public (where generally levels of knowledge are low), as being more knowledgeable may be a result of self-selection of people, especially those interested in the topic (Ferrari et al. 2021).

In a recent study in the USA, based on a survey among 2000 respondents, 75% of respondents favoured labelling of GE food while 41% was undecided about the risks and benefits of GE, while the remainder was about equally divided between accepting and opposing GE food (Lindberg et al. 2023). Other studies referred to in Lindberg et al. (2023) indicated that about half of the consumers have little knowledge of GE (one of those studies also noted that Americans are more supportive of GE than people in European countries). The relatively few not seeing a need for labelling appeared to perceive GE as equivalent to non-GE products and showed a self-reported higher level of knowledge and more trust in government regulators and industry. People opposed to GE food and favouring labelling showed more trust in NGOs, had more ethical concerns about technology and doubts about risks (perceiving them as high) and benefits, or considered benefits as unequally shared. Overall, Lindberg et al. (2023) found no clear relationship between labelling attitudes and specific values or concerns, probably because a large part of respondents favoured GE labelling. However, a broadly shared notion was the right to be informed.

In the UK, a study by the Food Standards Agency (FSA 2021) on consumer perceptions of GE food showed that 42% of respondents (out of 2,066 in total) did not know about GE food or confused it with GM food. The more knowledge respondents had, the more accepting they were of GE. GE food was found to be more acceptable (and GE plants more than GE animals) than GM food. They were mostly concerned about human and animal safety. Most respondents considered it would be appropriate to differentiate regulation between GE and GM, but also felt that, initially, the same level of scrutiny should be adopted. There was also concern that large corporations would undermine consumer benefits to maximize profits. Crucially, respondents felt that GE food should always be accompanied by information towards consumers indicating the presence of GE ingredients.

In a Norwegian study (GENEinnovate 2020), Norwegian consumers appeared to be quite knowledgeable about GM, but only half of the respondents had heard about GE. Again, the knowledge was important for accepting GE products. Overall, the respondents were positive about GE if it had a sustainable (such as decreasing the use of pesticides) or societal benefit. Many were concerned about potential risks, but products approved by the Norwegian authorities for the Norwegian market were associated with high confidence in health and the environment. Consumers were unwilling to pay a higher price for GE-modified food, even if had additional benefits. Finally, consumers wanted information on the product traits (including which technique was used and what kind of trait was modified) which would make it easier for them to choose.

The general trend in consumer studies is that the majority of consumers have limited knowledge of GE techniques and that GE acceptance increases with more understanding. Therefore, additional information might be provided on a potential GE label (such as a modified trait) to increase consumer acceptance. There was also a tendency to label GM and GE separately, as these techniques were seen as distinct from each other. However, GE techniques were rarely seen as equivalent to “natural” products, and the majority also was in favour of clear labelling, so an informed choice could be made. This further supports clear informative labelling towards consumers.

### Variety names and production chains (increasing food transparency towards consumers)

Transparency about the breeding history of a product can also be offered to consumers by providing the plant variety name, instead of specifically labelling NGT-1 products. Under UPOV DUS guidelines, each variety has a unique name, called a plant variety denomination (PVD). The name outlines the variety rights; i.e., it remains associated with the variety, even after the Plant Breeders’ Rights have expired. In the EU, the use of the PVD is mandatory when breeders, producers, plant nurseries, etc., offer or sell propagating materials, thus providing transparency, but it is not mandatory for the final step in the food chain, the selling of the harvest of plant varieties to consumers.

Using the name of a variety by distributors is therefore common in the production chain up till the supermarket, and often specific products are ordered for cultivation by the supermarket. Thus, the supermarket knows what varieties are being sold. Providing the variety name to the consumer as extra information would be relatively straightforward by requesting the information from distributors. This could provide food transparency to consumers through providing varieties names, without singling out NGT-1 varieties.

Differences exist among countries in whether variety names are provided directly to consumers. Some products in supermarkets are sold under a variety or brand name, but the distinction between these two is not clear to consumers. For example, potatoes are commonly listed under their variety names. Retailers use this to entice customers to pick potatoes with a different texture or taste and therefore also a different price (FSA Press Release 2003). Seed potatoes, in contrast, are always required to be labelled with the variety name to provide transparency to growers (European Commission 2002). Other vegetables are not commonly sold under their variety name.

Apples (and pears) are typically listed under their variety name, but there are some caveats. “Elstar” apples in the supermarket can refer to several (mutant) varieties essentially derived from the original Elstar variety, and each of these varieties has Plant Breeders' Rights, but their names (e.g. Elshof, Red Elstar) are not used towards consumers. Furthermore, “Golden Delicious” apples (a variety that is over 100 years old) come from various sources and mutant lines of trees. Next to variety names, brand names are also used as they offer more control over the production characteristics, volume, and price. For instance, with respect to apples, Pink Lady® is registered in more than 80 countries and is used to market several varieties, including “Cripps Pink”, “Sekzie”, “Rosy Glow”, “Ruby Pink”, and “Lady in Red” (Martínez López 2021). These names can be used to inform on breeding history of the variety.

In the UK, the packages of strawberries in supermarkets such as Tesco contain the name of the variety and the producing farm. In the Netherlands, this is not customary, but varieties are sometimes sold under a name, including “Sweet Eve” raspberries (Berryworld) and “Calinda” and “Lambada” strawberries (Fresh Forward). Sometimes, brand names of oranges (e.g. Sunkist®), mandarins (e.g. Cuties®), or speciality tomatoes (extra small or sweet) are also provided.

To conclude, variety and brand names may facilitate transparency in breeding history, at least to those who have knowledge of plant varieties. These names are currently mentioned for some fruits and vegetables, often in connotation with a buying incentive (e.g. different textures of potato varieties, or sweetness/texture levels of apples). It is, however, currently not common practice to give variety names for all products in supermarkets, although this information is available between supermarkets and distributors.

### Limitations

In the European Commission’s proposal on NGTs, downstream NGT-1 labelling will depend on a paper trail (or advanced techniques such as blockchain), since, in some cases, determining if a product has been achieved through conventional means or NGTs will be difficult if not impossible through molecular methods (ENGL 2023). This system may function well if there is trust among parties in the production chain, but it will not account for the unintended presence of admixtures, and it will reach a practical limit of use when varieties or products thereof are purposely mixed during processing or in final products. The issue of adventitious presence may mean that a limit of admixture may have to be defined, below which no labelling is necessary, similarly to the GM upper limit of 0.9%.

Purposely mixed products, such as wheat flour, may at some point get a “may contain” (or even a “may consist of”) label, not because of unforeseen admixture in the production chain (e.g. in the case of allergens), but because of a foreseen mixture of several varieties to obtain a predefined end quality. This mixing process cannot be easily quantified. In the case of bread wheat varieties used to produce ready mixes for bakeries, the exact mix is modified each year based on the characteristics of the wheat grains of various varieties, produced in different locations in that specific year. Vegetables and fruits are often sold as unit product and/or under a variety name, in which case this problem is not likely to occur.

To solve the issue of lack of transparency from anti-GMO critics, the retailers can provide information on the variety sold directly to the consumers. However, supermarkets and other retailers might be unwilling to share this information since competitors may gain insight into their marketing strategies. Even with the variety/brand name, customers might still not know what it entails unless they acquire additional (online) information and/or possess specific knowledge.

## Discussion and conclusions

Food labelling increases the freedom of choice of consumers. In the EU, food packages are required to contain information such as the ingredient list and the possible presence of allergens. The EU also has mandatory labelling of GMOs, and GM crop cultivation is very limited. Next to mandatory information and labels, voluntary labels also exist, such as the Fairtrade and Rainforest Alliance labels. These are determined nationally, but need to comply with established regulations set by intergovernmental bodies.

In the recent proposal of the European Commission on the regulation of plants obtained by certain NGTs, NGT-1 category plants are not labelled and transparency is guaranteed by mandatory labelling of NGT-1 seed and PRM in the production chain. In addition, NGT-1 varieties will be included in a public register, and these varieties are excluded from organic farming and breeding. An NGT-1 variety register may be similar to or even might be integrated into lists already available in systems such as organic farming or VCU lists. The aforementioned positive list with vegetable varieties is based on transparency from the producers, and it is used in organic farming for excluding CMS varieties. The proposal for a new VSCU would have the added value of providing sustainable characteristics for vegetables and fruits. These traits are also interesting for GE products and can be included in an NGT-1 register.

Non-GMO campaigners argue that freedom of choice for consumers is not guaranteed in the present proposal, despite a register for NGT-1 plants, and also demand clear labelling on the end product. However, this would lead to contested labelling of NGT-1 varieties, while conventional products with the same modifications remain unlabelled. Furthermore, detecting modifications and determining if they have been achieved by NGTs or conventionally would not be possible and would depend on a paper (or electronic) trail.

Two considerations need to be made regarding the potential labelling of NGT-1 products. First, labelling adds additional costs and needs to be proportional. An NGT-1 label will not be informative regarding food safety (cf. this information is also not supplied for products made by random mutagenesis) and only refers to the breeding technology used. As an alternative, information on the technology used can be proportionally supplied in the production chain by positive lists, in the way these are already currently used in organic farming and breeding. Second, in case it would be decided that category NGT-1 products need to be labelled in the EU, adding information on beneficial traits to the label could lead to greater consumer acceptance. Increasing knowledge of NGTs in plant breeding can also positively influence consumer acceptance.

To conclude, different scenarios are possible regarding increasing the consumer transparency in the production chain for plant varieties produced using NGTs. As an alternative to mandatory labelling, the discussed positive lists in breeding and farming may be used, or the variety names can be provided at the retailer. Both methods would contribute to an informed choice of the consumer, while not discriminating against NGT-1 varieties. In the case of mandatory labelling, a positive aspect can be added (similar to NGT-2 varieties) by listing potential beneficial modified traits. As consumer studies on GE food indicate, providing this information on the modified trait can lead to increased consumer acceptance.

## Data Availability

Data availability is not applicable. No new data have been generated.
